# The prevalence of pseudoexfoliation syndrome in Pakistan. Hospital based study

**DOI:** 10.1186/1471-2415-6-27

**Published:** 2006-06-22

**Authors:** Rashad Qamar Rao, Tariq Mehmood Arain, Muhammad Ali Ahad

**Affiliations:** 1 Department of Ophthalmology, Bahawalpur Victoria Hospital, Bahawalpur, Pakistan; 2 Department of Ophthalmology, South Wing St Thomas' Hospital, London SE1 7EH, UK

## Abstract

**Background:**

Pseudoexfoliation syndrome (PXS) is the most common identifiable cause of secondary glaucoma, the prevalence of which varies considerably among different ethnicities. The aim of this study was to assess the prevalence of pseudoexfoliation syndrome in Pakistan.

**Methods:**

A prospective study conducted in the period from January 2003 to June 2004 in a teaching hospital serving a population of about 7.6 million. 1860 patients aged 45 or above attending the general ophthalmic clinics were recruited for this study. A detailed evaluation including ophthalmic and general history, slit lamp biomicroscopy, intraocular pressure measurement, gonioscopy and dilated eye examination was performed on all patients.

**Results:**

120(6.45%) subjects were found to have PXS with a male to female ratio of 1.5:1. All 120 (100%) cases were bilateral and 48(40%) patients had high intraocular pressure.

**Conclusion:**

To the best of our knowledge, this is the first study conducted in a Pakistani population to determine the prevalence of PXS. The prevalence rate of 6.45% is similar to other studies conducted in south Asia, however all cases were bilateral and quite a high percentage of patients had high intra-ocular pressure.

## Background

Pseudoexfoliation syndrome (PXS) is characterized by the deposition of a distinctive fibrillar material in the anterior segment of the eye and was first described in 1917 by Lindberg [1]. It is frequently associated with open angle glaucoma, known as pseudoexfoliation glaucoma, which is the most common identifiable form of secondary open angle glaucoma worldwide [2]. Despite extensive research, the exact chemical nature of the fibrillar material is unknown. It is believed to be secreted multifocally in the iris pigment epithelium, the ciliary epithelium, and the peripheral anterior lens epithelium [3]. The material moves into the aqueous humor and is carried to the trabecular meshwork, following the normal flow. Obstruction of the trabecular meshwork by this fibrillar material and pigment causes elevation of the intraocular pressure (IOP) leading to glaucoma [4].

PXS is now suspected to be a systemic disorder and has been associated preliminarily with stroke, systemic hypertension, and myocardial infarction [5]. In fact pseudoexfoliative like material has been found in lungs, skin, liver, heart, kidney, gallbladder, blood vessels, extra ocular muscles and meninges [6].

PXS is rarely seen before the age of 50, and its prevalence increases markedly with age [7]. Although it occurs in virtually every area of the world, a considerable racial variation exists. In the Framingham study, prevalence of PXS was found to be1.8% [8]. In another study of subjects over 60 years in various ethnicities, prevalence rates ranging from 0% in Greenland Eskimos to 21% in Icelanders were noted [9]. In northern/western European countries including England, Germany, and Norway prevalence of 4.0%, 4.7%, and 6.3% have been reported respectively [10].

Although epidemiological studies of PXS have been done in South Asia [11,12], but there is no data available on prevalence of PXS in Pakistan. This study was performed to determine the prevalence of PXS in Pakistan.

## Methods

After approval from Hospital Ethics Committee, cross-sectional hospital based study was performed at Bahawalpur Victoria Hospital, (BVH) Bahawalpur, Pakistan. BVH is a regional tertiary care centre and university hospital of Bahawalpur division that serves a population of 7,635,591 people. (PopulationCensus Organization 1998-census, Government of Pakistan) All the patients who were 45 years of age or older presenting to eye out patient department from January 2003 to June 2004 were asked to enrol for this study. After informed consent the patients underwent complete ophthalmic evaluation. This included complete ophthalmic and general history, best corrected visual acuity, slit lamp examination, applanation tonometry and gonioscopy. The patients were then dilated and slit lamp examination of the lens and fundus was carried out. The patients who were found to have narrow or occludable angles on gonioscopy were first offered laser iridotomy treatment. The rest of the evaluation was then deferred to a later date. An angle was considered occludable if the pigmented trabecular meshwork was not visible in 180 o or more of the angle. For IOP measurement, only the higher IOP between the two eyes was considered. Any IOP of more than 22 mm Hg was considered as high and only if the pressure was again high after 10 minutes of wait and tested by a second examiner on a different slit lamp. The criterion used to diagnose PXS was the presence of pseudoexfoliation material on one or more anterior segment structures. Since the presence of pseudoexfoliative material on lens is the most consistent and prominent feature of PXS, so to prevent under estimation of the prevalence, all subjects who were psuedophakic or aphakic in any eye were excluded from the study. Also noted were demographic details, reason of attendance and presence of any systemic disease. Since the aim of the study was prevalence of PXS in a given population so no attempt was made to determine the prevalence or incidence of glaucoma in PXS patients.

Statistical analysis was performed using Statistical Package for Social Sciences (SPSS version 12.0, Chicago IL). Chi square test was used to compare discrete variables and Mann Whitney test was used to compare ordinal data.

## Results

1860 patients were recruited for the study. There were 1040 males and 820 female with a male female ratio of 1.27:1. Demographic details and the reason of attendance to the eye clinic are shown in Table 1. The stratification of age is shown in Table 2.

**Table 1:** Demographic details and reason of attendance to the eye clinic

	All Patients n = (1860)	Patients with PXS N = (120)
Age in years	45–87 (63.6)	51–84 (72.2)
Male to Female ratio	1.2–1	1.5–1
Mean Vision	6/18	6/12
Prevalence of diabetes	10.2%	10.8%
Prevalence of high blood pressure	13.4%	16.6%
**Reason of Attendance**		
Cataract	32.3%	52.5%
Ocular surface/lid disorders	23.7%	8.3%
Age related macular degeneration	11.3%	15.0%
Diabetic retinopathy	8.6%	5.0%
Glaucoma	8.2%	12.5%
Uveitis	3.0%	0.0%
Retinal vascular diseases	3.0%	5.0%
Neurophthalmological Problems	2.9%	0.8%
Hereditery Retinal diseases	1.7%	0.0%
History of Infectious corneal ulcers	1.3%	0.8%
Others	4.1%	0.0%

**Table 2:** Age distribution of studied population

**Age range**	**No. of subjects**	**Percentage**
45–59 years	714	38.40%
60–69 years	456	24.50%
70 and above	690	37.10%
Total	1860	100%

120 patients were found to be having PXS with an overall prevalence of 6.45%. The salient clinical features of the patients with PXS are shown in Table 3. All cases were bilateral though asymmetrical. The mean age of patients with PXS was 72.2 years, which was significantly higher compared to 63.6 in whole examined population (p < 0.0001)

**Table 3:** Clinical features of the patients with pseudoexfoliation syndrome

Clinical Features	n = (120)
Pseudoexfoliation of lens	118 (98.3%)
Flakes on Iris margin	98 (81.7%)
Poor pupil dilation	80 (66.7%)
Iris abnormalities	76 (63.3%)
Flakes on trabecular meshwork	68 (56.7%)
High intraocular pressure	48 (40%)
Zonular weakness	30 (25.0%)
Bilateral disease	120 (100%)
Occludable angles	5 (4.1%)

There were 72 males and 48 females with a male to female ratio of 1.5:1. The prevalence of PXS was 6.92% in males and 5.8% in females and this difference was not significant. The prevalence of PXS increased with the increasing age as shown in Table 4.

**Table 4:** Prevalence of pseudoexfoliation stratified according to age groups.

**Age range**	**Number**	**percentage**	**prevalence**
50–59	5	4.2%	0.70%
60–69	25	20.8%	5.48%
70 & above	90	75%	13.04%
Total	120	100%	6.45%

As shown in table 4, 75% of the patients with PXS were 70 years old or above. The prevalence was 2.6% in patients less than 70 years of age, which increased to 13.04% in patients 70 years old or older. (*p *= 0.001). High IOP (more than 22) was noted in 48 patients (40%). It was also noted that prevalence of high IOP increased with increasing age. The mean age of patient with PXS and high IOP (n = 48) was 74.2 years compared to 71 years in patients with normal IOP (n = 72) *p *= 0.02. Occludable angles were seen in 5 patients (4.1%) with PXS compared to 40 patients (2.29%) with out PXS. Although the occludable angles were more common in patients with PXS but the difference was not statistically significant and this difference could also be attributed to the age which was significantly higher in PXS group.

## Discussion

The reported prevalence rate of PXS syndrome in different populations shows extensive variations, 0% in Eskimos [9], 1.8% in the United States [8], 5–25 % in the Scandinavian countries [9], and 38% in Navajo Indians [13].

This variation is combination of true differences due to racial difference, the age and sex distribution of the population group examined, the criteria used to define examined populations that is, persons over a certain age, patients taken from eye clinics, patients with cataracts, and glaucoma patients. The prevalence of PXS increases progressively among the following groups: 1) the general population; 2) persons over age 50; 3) ocular hypertensives; 4) glaucoma patients; 5) glaucoma patients admitted to the hospital.

This study is first study of its kind from Pakistan. Our examined population was recruited from the eye clinic and we found the prevalence to be 6.45%. This is quite similar to 7.4% in a similar hospital based study from India [14] and 6% in south Indian population based survey [15] and slightly lower than in neighboring Iran where the prevalence was found to be 9.6% [16]. These geographic distribution patterns may perhaps be explained either by regional gene pools or by environmental influences. However it should be noted that prevalence in our study was significantly higher from two large epidemiological studies from South India [11,12] and it could be attributed to design of the study which was different in our study.

We did not notice any sex predilection; however the prevalence was slightly higher in males. As expected and reported in other studies [17-19] the prevalence increased with increasing age reaching 13.04% in patients over 70 years of age.

An unexpected finding was a high percentage (40%) of the subjects with PXS had high intraocular pressure. Most of the studies have shown prevalence of high intraocular pressure with or without glaucoma to be in between 22% to 30% [20-22]. It is quite possible that this high rate in our study may be due do the design of study which was hospital based.

We also noted that the PXS was bilateral in 100% of the cases, which is not documented by any study, but as shown by Prince et al and Speakman et al, the disease is invariably bilateral pathologically [23,24]. In our study, 30 eyes of the total 240 eyes with PXS had flakes on the iris only. The presence of flakes on the iris can be missed easily particularly in light colored eyes, so either the darker color of the iris makes it easily recognizable or south Asian people truly have high prevalence of clinically bilateral PXS [15] could be the possible explanation.

## Conclusion

In conclusion we found the prevalence of PXS to be 6.45% in a Pakistani population. This rate is similar to other studies conducted in south Asia, however all cases were bilateral and quite a high percentage of patients had high intra-ocular pressure.

## Abbreviations

PXS: pseudoexfoliation

IOP: intra-ocular pressure

BVH: Bahawalpur Victoria Hospital

## Competing interests

The author(s) declare that they have no competing interests.

## Authors' contributions

RQR conceived of the study and carried out clinical examination and data collection. TMA participated in clinical examination and data collection. MAA designed the study, carried out statistical analysis and drafted the manuscript. All authors read and approved the final manuscript.

## Pre-publication history

The pre-publication history for this paper can be accessed here:


